# Communication Tools Used in Cancer Communication with Children: A Scoping Review

**DOI:** 10.3390/cancers14194624

**Published:** 2022-09-23

**Authors:** Noyuri Yamaji, Daichi Suzuki, Maiko Suto, Kiriko Sasayama, Erika Ota

**Affiliations:** 1 Global Health Nursing, Graduate School of Nursing Science, St. Luke’s International University, 10-1 Akashi-cho, Chuo-ku, Tokyo 104-0044, Japan; 2 Department of Nursing, Faculty of Health and Medical Sciences, Kanagawa Institute of Technology, 1030 Shimo-ogino, Atsugi 243-0292, Japan; 3 Department of Health Policy, National Center for Child Health and Development, 2-10-1 Okura, Setagaya-ku, Tokyo 157-8535, Japan; 4 Global Health Nursing, International University of Health and Welfare, 4-3 Kozunomori, Narita, Chiba 286-8686, Japan; 5 Tokyo Foundation for Policy Research, Roppongi Grand Tower 34F, 3-2-1 Roppongi, Minato-ku, Tokyo 106-6234, Japan

**Keywords:** cancer, children, communication, communication tools, scoping review

## Abstract

**Simple Summary:**

Despite the potential benefits of effective communication, telling children about unpredictable and life-threatening conditions such as cancer is challenging. This scoping review aimed to map the potential communication tools for children with cancer, their families, and healthcare professionals. We found 25 studies and 21 communication tools. Communication tools might support children to improve their knowledge and psychological outcomes. However, we found a lack of communication tools that were (1) accessible and validated, (2) designed for healthcare professionals, (3) targeted children, families, and healthcare professionals, and (4) were designed to meet the needs of children and families. This review identified areas for further research.

**Abstract:**

Background: Although communication tools might guide healthcare professionals in communicating with children about cancer, it is unclear what kind of tools are used. This scoping review aimed to map the communication tools used in cancer communication among children with cancer, families, and healthcare professionals. Methods: A comprehensive search using PubMed (including MEDLINE), Embase, CENTRAL, PsycINFO, and CINAHL was conducted on 1 August 2021. We mapped communication tools and their impacts. Results: We included 25 studies (9 experimental studies and 16 feasibility studies) of 29 reports and found 21 communication tools. There was a lack of communication tools that were (1) accessible and validated, (2) designed for healthcare professionals, (3) targeted children, families, and healthcare professionals, and (4) were designed to meet the needs of children and families. Experimental studies showed that the communication tools improved children’s knowledge and psychological outcomes (e.g., health locus of control, quality of life, self-efficacy). Conclusion: We mapped communication tools and identified areas that needed further research, including a lack of tools to guide healthcare professionals and share information with children and families. Further research is needed to develop and evaluate these communication tools. Moreover, it is necessary to investigate how communication tools support children, families, and healthcare professionals.

## 1. Introduction

Worldwide, approximately 300,000 children under the age of 14 are diagnosed with cancer annually [1]. Cancer is a life-threatening condition [2] and children with cancer face health threats, and sometimes have stressful and traumatic experiences [3,4]. Effective communication helps children prepare for treatments and the future [5,6,7,8], while lack of information gives children a psychological burden and makes them distrustful of their parents and healthcare professionals (HCPs) [9,10].

Effective communication promotes potential benefits. However, telling children about cancer is a daunting challenge for families and HCPs because it is a life-threatening condition [11]. Several factors within the triad of children with cancer, their families, and HCPs sometimes obstruct communication with children. Many children need to receive understandable information openly and honestly [5,12,13], while they also desire to maintain a sense of hope [7,14]. Communication with children is affected by parents’ own understanding and emotional response to the diagnosis. If parents are too shocked and unable to grasp children’s diagnosis or believe that children cannot understand and admit their diagnosis, miscommunication or misinterpretation of information might follow [9,15].

HCPs require excellent skills to adapt to fit the child and family’s needs [12,16,17]. However, HCPs’ emotional and mental strain, insufficient time to communicate, and lack of confidence in communication skills were pointed out [18].

Recently, the complexity of disclosing prognosis to children has been recognized, leading to a renewed question of how and when to disclose it [11]. Although there are a few previous guides on general communication methods [12,19,20], previous studies have pointed out that more specific guides that consider children’s developmental stages and psychological status to improve communication with children are needed [9,11]. Communication tools developed with this consideration might help and guide HCPs in conveying the bad news of cancer-related information to children. However, it remains unclear how communication tools will be used to communicate with children with cancer and their families, and how they will impact their health outcomes. Therefore, this scoping review aimed to systematically map the following questions: (a) What communication tools are used in cancer communication with children, (b) are the communication tools available and how do children use these tools, (c) how are these communication tools validated and evaluated, and (d) how do these communication tools affect health outcomes?

## 2. Materials and Methods

### 2.1. Study Design

We conducted a scoping review and reported following the Preferred Reporting Items for Systematic reviews and Meta-Analyses extension for Scoping Reviews (PRISMA-ScR) Checklist [21]. Communication tools were defined as items or resources that help HCPs (e.g., physicians, nurses, child life specialists) and families (e.g., parents, caregivers) talk with children about their illness, including life-threatening conditions, and improve conversation among children, caregivers, and HCPs. We included any communication tools such as a checklist, book, brochure, computer game, playing with a doll, and drawing. Our protocol was also drafted following the preferred reporting items for systematic reviews and meta-analyses Protocols (PRISMA-P) [22] and published to the journal of BMJ open [23].

### 2.2. Eligibility Criteria

We have decided on the eligibility criteria following the PCC (Population/Concept/Context) framework guided by the Joanna Briggs Institute [24]. We included studies which researched (Population) children between 2 and 18 years of age diagnosed with any type and stage of cancer; (Concept) communication tools to provide information related to cancer to children with cancer, including cancer diagnosis, life-threatening conditions, symptoms, treatments, prognosis, and psychosocial effects; and (Context) in the healthcare setting to communicate with children about cancer. We also included peer-reviewed original primary articles without limitations such as study design and languages. If we could not separate the data of children with cancer from adults, we included the studies in which over 80% of the population was under 18. We excluded the studies investigating tools without cancer-related information or educational elements (e.g., play interventions, symptom management), and interventions featuring distraction techniques to divert the child’s attention from harmful stimuli (e.g., during painful procedures).

### 2.3. Information Sources and Search

We searched the following electronic bibliographic databases: PubMed (including MEDLINE), Embase, the Cochrane Central Register of Controlled Trials (CENTRAL), PsycINFO, and CINAHL on 1 August 2021 with no date/time, language, document type, and publication status limitations. The search strategies were developed by assistance of a medical information specialist, including terms relating to PCC (Supplementary File). Additionally, we checked reference lists of included articles and relevant reviews for this study [25]. We followed the Cochrane Handbook [26] and Cochrane’s MECIR [27] to conduct the search, PRISMA-S [28], PRISMA-ScR [21], PRISMA guideline [29] to report the search, and PRESS guideline while peer-reviewing the search strategies [30].

### 2.4. Selection of Sources of Evidence

**Figure 1:**
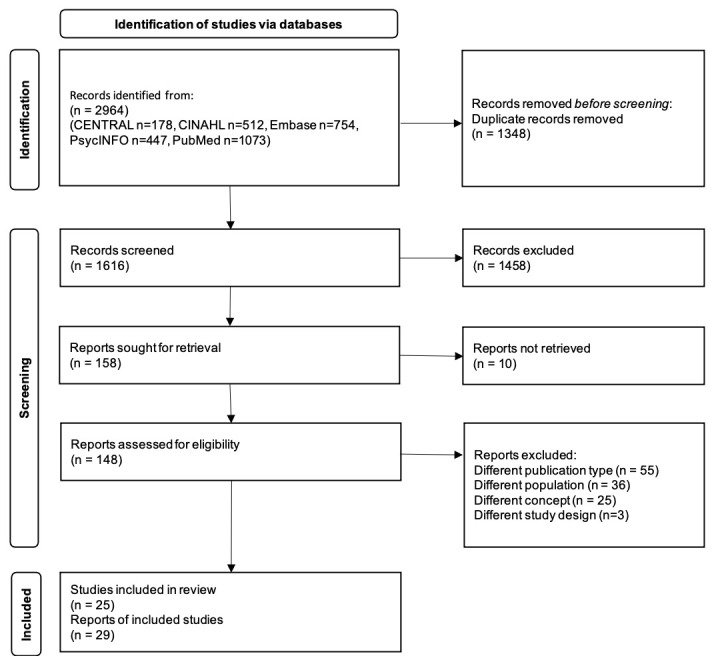
Process of selection studies.

Search results were de-duplicated in EndNote X7 and imported to Rayyan, a web application, to screen the eligible studies [31]. Process of selection of studies has been shown in PRISMA flow diagram (Figure 1). Two or more reviewers (NY, DS, MS, and KS) independently screened the eligible studies following the Preferred Reporting Items for Systematic Reviews and Meta-Analyses extension for scoping reviews (PRISMA-ScR) [21]. We discussed disagreements and resolved them.

### 2.5. Data Charting and Data Item

For the included studies, two or more reviewers (NY, DS, MS, and KS) independently charted the characteristics of included studies and communication tools into the data-charting forms developed by MS Excel for this study.

### 2.6. Critical Appraisal of Individual Sources of Evidence

Since this review aimed to map the existing evidence, we did not assess the critical appraisal of individual studies [21,32].

### 2.7. Data Synthesis

We summarized the characteristics of included studies, including the study design, the purpose of the study, settings, populations, intervention or concept, and broad findings. The characteristics and impacts of communication tools were mapped into Table 1 and Table 2.

## 3. Results

### 3.1. Selection of Sources of Evidence

Of 2964 citations retrieved, 1348 records were removed to avoid duplicates, and 1616 records were screened. We excluded 1458 records which did not meet the inclusion criteria, and 148 full texts were assessed, excluding 10 articles that were not retrieved. Finally, we included 25 studies out of 29 reports (Figure 1).

### 3.2. Characteristics and Results of Sources of Evidence

The characteristics and findings of the included studies are shown in Table 1. Nine studies assessed the effects of the communication tools [33,34,35,36,37,38,39,40,41]. Sixteen feasibility studies focused on developing the tools and assessed their feasibility [42,43,44,45,46,47,48,49,50,51,52,53,54,55,56,57]. The included studies were conducted in 11 countries involving more than 1562 participants, including children with cancer, their parents, and HCPs. Ten studies out of twenty-five were conducted in the USA [33,34,36,41,45,48,49,50,56,57], and other studies were researched in various countries (two studies: Brazil, Canada, and multiple countries [37,42,44,53,54,55]), one study: China, Germany, Iran, Norway, Pakistan, Sweden, and Turkey [35,38,39,40,43,47,52]). Two studies did not specify the country setting [46,51]. These studies were published constantly from 2002 to 2021. Eight studies researched the same tools as other studies [38,52,54,55,58,59,60,61]. There was a variety of interventions, such as computer games (seven studies) [35,37,39,40,45,48,57], play therapy (five studies) [42,44,51,53,56], computer-based communication tools (three studies) [43,47,50], CD-ROM (three studies) [33,34,36], videos (two studies) [41,46], and brochures (one study) [49].

**Table 1:** Characteristics of included studies.

Author, Year	Study Design	Purpose of the Study	Study Setting	Study Participants	Intervention or Concept	Study Findings
Artilheiro, 2011 [42]	Exploratory descriptive study	To describe the use of therapeutic play (TP) in the preparation, and to identify manifestation during TP session	At the oncology outpatient department from Hospital Infantil Darcy Vargas, São Paulo in Brazil	Children (3–6 years old) who submitted to chemotherapy in the outpatient department (*N* = 30)	Therapeutic play during the chemotherapy	TP facilitated child more positive behaviors, cooperating with the procedures, working with professionals (93.3%), with relaxed posture (93.3%), establishing a bond of trust with the professional (76.6%), and smiling while playing (70%)
Arvidsson, 2016 [43] Baggott, 2015 [58]	User-experience design	To redesign Sisom and validate and adapt it for use in a Swedish population of children with cancer	Sweden	Swedish translators (*n* = 4), Norwegian translators (*n* = 2), pediatric nurses working with the care of children with cancer (*n* = 2), and healthy children (*n* = 2)	Interactive computer-based assessment and communication tool for children with cancer	Sisom was revised following the participants’ feedback
Beltran, 2013 [57]	Qualitative study	To assess the effect of the video games	Children’s Cancer Hospital, Texas in United States of America (USA)	Children with cancer and survivors (9–12 years old) who have a high risk of obesity (*N* = 28)	Escape from Diab and Nanos warm: Invasion from Inner Space are videogames about preventing obesity	Two themes emerged to guide future modifications of the game: difficulty with the energy balance and meal selection and endings in which the character died made them sad
Bisignano, 2006 [33]	Randomized controlled trial (RCT)	To assess the influence of a developmentally specific compact disc read-only memory (CD-ROM) intervention	Oncology clinic at a large urban medical center in USA	Children (7–18 years old) scheduled for IV procedures (*N* = 30)	CD-ROM designed to help children learn about the medical procedure	CD-ROM reduced threat appraisals and improved cognitive restructuring coping. However, there was no clear evidence of fear, behavioral distress, or pain
Dragone, 2002 [34]	RCT	To assess the effect of CD-ROM compared with the book	District of Columbia, Virginia, and Ohio in USA	Children with leukemia (4–11 years old) (*N* = 14 + 7), and their families (*N* = 16 + 8)	CD-ROM designed to improve children’s feelings control and understanding of leukemia	CD-ROM was associated with an increase in health locus of control CD-ROM was a useful, engaging, and empowering tool for children with leukemia
Fazalniya, 2017 [35]	RCT	To investigate the effect of an interactive computer game	One hospital in Iran	Children with cancer (8–12 years old), were receiving treatment and undergoing at least 4 months of chemotherapy (*N* = 64)	The intervention program included an educational-entertainment computer game named “The City of Dreams” which was developed by authors.	The computer game increased quality of life (QOL) in children with cancer
Frygner-Holm, 2020 [44]	Mixed method	To develop and evaluate the feasibility and acceptability of a pretend play intervention	Three universities in Sweden, USA, and Germany	Children with cancer (4–10 years old) (*N* = 5)	Pretend play to support children’s communication, self-efficacy, and coping ability in the care setting	Pretend play improved self-efficacy Pretend play increased or equal QOL There were no adverse events or increased worrying
Fuemmeler, 2020 [45]	Feasibility study-quasi-experimental single-group pretest/posttest design	To describe the development and initial feasibility evaluation of the intervention	Two pediatric oncology clinics, Duke University and Chapel Hill in USA	Pair of pediatric cancer survivors (12–17 years old) and their parents (*N* = 16)	App-based game “Mila Blooms” that promotes healthy eating and physical activity among adolescent survivors of childhood cancer	Mila Blooms holds promise for promoting health behavior change
Greenspoon, 2019 [46]	Feasibility study	To assess (1) the understandability, actionability, and readability of the video; (2) patient and caregiver perceptions, knowledge, and interest in FP; and (3) satisfaction with a patient education video	At oncology clinics at a pediatric center and an adult center (not specified country setting)	Patients (13–39 years old) after minimum 1 month from diagnosis (*N* = 108) (pediatric center: *n* = 30; average age, 17 years; adult center: *n* = 78; average age, 30 years) and 39 caregivers or partners (pediatric center, *n* = 30; adult center, *n* = 9)	Whiteboard video to explain egg cryopreservation to patients and families	Video can build knowledge and encourage discussions about infertility
Jones, 2010 [36]	RCT	To develop and assess the effects of developed CD-ROM compared with Handbook	Four pediatric oncology programs, Los Angeles (California), District of Columbia, Hershey (Pennsylvania), and New York City in USA	Children with solid tumors (12–18 years old), had being treated or within 3 years of treatment (*N* = 185) However, the final sample consisted of 65 children (CD-ROM: *n* = 35, Handbook: *n* = 30)	CD-ROM to educate adolescents about their cancer	The CD-ROM improved health locus of control and got high marks from adolescents with cancer, their families, and healthcare providers There was no significant difference in QOL, self-efficacy, coping style, and cancer knowledge between the CD-ROM and control group
Kato, 2008 [37] Beale, 2006 [60] Beale, 2007 [59] Kato, 2006 [61]	RCT	To determine the effectiveness of a video-game intervention	34 cancer treatment centers in USA, Canada, and Australia	Youth with cancer (13–29 years old), were receiving treatment and were expected to remain on treatment for at least 4–6 months. (*N* = 375) Age 13–18 (*N* = 324, 87.3%) and age 19–29 (*N* = 47, 12.7%)	Re-Mission was designed to be a learning environment that motivates, guides, and supports the learning of a set of behavioral objectives related to self-care during treatment for cancer. (http://www.re-mission.com/) (accessed on 20 September 2022)	The video-game intervention increased adherence, self-efficacy, and knowledge compared with no cancer-related game There was no clear difference in self-report measures of adherence, stress, control, or QOL between the intervention group and the control group
Kock, 2015 [47]	Feasibility study-cross-sectional study	To increase compliance with follow-up examinations using a reminding service	Two locations: the University Medical Center in Lübeck and the University Hospital Hamburg-Eppendorf in Germany	Former patients from the age of 15 and their relatives (*N* = 22)	Mobile application to provide the information on late effects of childhood cancer	The application is expected to increase the awareness for follow-up visit. The Aftercare App will help former patients structure their long-term follow-up care and survey the key information they require
Kurt, 2013 [38]	Before-after controlled study	To determine the effects of Re-Mission video game	At two hospitals, Istanbul in Turkey	Adolescents with cancer (13–18 years old) (*N* = 61)	Re-Mission was designed to be a learning environment that motivates, guides, and supports the learning of a set of behavioral objectives related to self-care during treatment for cancer (http://www.re-mission.com/) (accessed on 20 September 2022)	Re-Mission video game positively affected QOL, while no significant difference between groups in the average scores of QOL in the first measurement
Li, 2011 [39]	A non-equivalent control group pretest–post-test, between-subject design	To examine the effectiveness of therapeutic play, using virtual reality computer games	One of the largest acute-care hospitals, Hong Kong in China	Hong Kong Chinese children hospitalized with cancer (8–16 years old) (*N* = 120)	30-minutes therapeutic play intervention by research nurse using virtual reality computer games daily (five days a week)	Therapeutic play using virtual reality reduced depression compared with the control group There was no clear difference in children’s anxiety between the intervention group and the control group
Linder, 2021 [48]	Feasibility study–qualitative study by interviews	To evaluate the feasibility and acceptability of a newly developed game-based symptom-reporting app	A children’s hospital in the USA	Children with cancer, (6–12 years old) were undergoing treatment (*N* = 19)	Game-based symptom-reporting app, “Color Me Healthy”	Study results support the preliminary feasibility and acceptability of the app
Murphy, 2012 [49]	Qualitative study by face-to-face interviews	To test the design, readability, likelihood to read, and overall opinion of a pediatric fertility preservation brochure	Children’s Cancer Center and All Children’s Hospital, Florida in USA	Children with cancer and survivors (12–21 years old) (*N* = 7), their parents (*N* = 11), and healthcare professionals (*N* = 6)	Two versions of gender concordant brochures on fertility for pediatric oncology patients and their parents	All female teens and parents preferred brochure version 1 The majority of parents preferred version 2, while the majority of male teens preferred version 1
O’Conner-Von, 2009 [50]	Qualitative study by interviews	To develop and validate an innovative, interactive web-based educational program	Pediatric oncology clinic at a university medical center in USA	Adolescents who had completed cancer treatment within the past 12 months (10–16 years old) (*N* = 4) and their parents (*N* = 5)	Web-based educational program to cope with cancer	The web-based educational program “Coping with Cancer” was developed and revised following adolescents with cancer and their families’ opinions
Pitillas, 2018 [51]	Qualitative study	To delineate a systematic approach for the use of play therapy (PT) among psychotherapists working within the field of pediatric oncology	Not reported	Children with cancer (3 years old) (*N* = 1)	Psychoanalytic PT depending on children’s needs and developmental stages	This article described the integrated play therapy in the psychological assistance of children with cancer The therapist may act as an informer, helping the child better understand her condition and its implications to help reduce uncertainty
Ruland, 2007 [52]	Qualitative study	To describe the process of development of the computer application, “SISOM”	The principal of a nearby elementary school in Oslo, Norway Design sessions were held at the “Adolescent Club Room” within the pediatric department in Norway’s National Hospital, Rikshospitalet, Oslo in Norway	Children in 4th (9 years old) and 6th (11 years old) grade (*N* = 50). The final group consisted of 12 children who worked in two separate design groups: one group of six 4th graders (9 years old) and one group of 6th graders (11 years old). Other children participated in other tasks	SISOM, a handheld, portable computer application to (1) help children with cancer aged 7–12 years old, communicate their symptoms/problems in a child-friendly, age-adjusted manner; and (2) assist clinicians in better addressing children’s experienced symptoms and problems in patient care	This study focused on the process of design development and described the importance of sharing insights from this collaborative design process
Sajjad, 2014 [40]	Experimental study	To measure the psychological symptoms in children with brain cancer and work on them through game therapy compared with control group	Three hospitals in Pakistan	Children with brain cancer (10–14 years old) (*N* = 76)	3D Graphical Imagery Therapy game on psychological signs of cancer patients fighting against brain cancer	3D Graphical Imagery Therapy game improved in self-conceptualization and has been effective for recovery from psychological illness related to a brain tumor
Sposito, 2016 [53]	Exploratory study with qualitative data analysis	To present the experience of using finger puppets as a playful strategy	At the pediatric oncology ward of a public teaching hospital in Brazil	Hospitalized children with cancer (7–12 years old), were undergoing chemotherapy treatment (*N* = 10)	Using puppets as a playful strategy during the interviews with hospitalized children with cancer	The use of the puppets, creatively and in accordance with the children’s motor, cognitive, and emotional development, showed benefits, such as allowing the children to freely express themselves; respecting their autonomy; and minimizing the hierarchical adult–child relationship
Tsimicalis, 2017 [54]	Single-site, descriptive, qualitative study	To produce a Sisom, interactive tool French version that is (1) clear, comprehensible, and understandable; (2) culturally and clinically meaningful; and (3) conceptually equivalent to the original version	At pediatric hospital, Montreal in Canada	Healthcare professionals who provided care in French to children with cancer (*N* = 5) Children with cancer (6–12 years old) (*N* = 10) and their parents (*N* = 10)	Interactive assessment and communication tool designed to provide children with a voice	Sisom was well received by participants who were forthcoming with input and suggestions for improving the French translations
Tsimicalis, 2018 [55]	Multisite descriptive study	To test the usability of Sisom	Three university-affiliated health centers in Canada	Children with cancer (6–12 years old), were received treatment or follow-up care at one of the study sites and their parents (*N* = 34)	Interactive assessment and communication tool designed to provide children with a voice	The majority of children liked Sisom and found Sisom easy to use, found it to help express their symptoms, and were satisfied with the aesthetics Some children provided suggestions for improvement to optimize Sisom use
Tyc, 2003 [41]	RCT	To determine whether a risk counseling intervention would increase knowledge and perceived vulnerability to tobacco-related health risks and decrease future intentions to use tobacco	St. Jude Children’s Research Hospital, Memphis in USA	Preadolescents and adolescents (10–18 years old), were previously treated for cancer (*N* = 103)	Educational video and risk counseling intervention was designed to be administered in a single session with periodic reinforcement of tobacco goals by telephone	Video and risk counseling intervention might improve knowledge, perceived vulnerability, and decreased intention at 12 months
Wiener, 2011 [56]	Pilot study, cross-sectional study	To learn how the game is being used in clinical settings and to gather information regarding the usefulness of ShopTalk	2009 American Pediatric Oncology Social Work (APOSW) annual meeting	Healthcare professionals (*N* = 110)	Therapeutic game to help youth living with cancer talk about their illness in a non-threatening way	ShopTalk appears to be a beneficial therapeutic tool in building rapport and identifying and discussing difficult issues with medically ill children

Footnote: the background is used for visibility. CD-ROM: compact disc read-only memory, TP: therapeutic play, PT: play therapy, QOL: quality of life, RCT: randomized controlled trial, USA: united states of America.

### 3.3. Synthesis of Results

The included studies showed 21 types of communication tools. Characteristics of communication tools are shown in Table 2.

**Table 2:** Characteristics of communication tools.

Author, Year	Contents	Mode/Type	Target Population	Developer	Access (e.g., Cost, Website, Article)	Usage Instructions	Evaluation or Validation of Communication Tool	Impact of Communication Tools on Health Outcomes and Outcome Measurements
**For children**								
Artilheiro, 2011 [42]	Therapeutic Play (TP) using a doll and other materials during chemotherapy such as: an intravenous device, cotton, syringe, needle, tourniquet, infusion pump, adhesive tape, and gauze, among others	Nurse-led TP during the chemotherapy	Not specified. However, it must be children with cancer	Not specified	Not reported	TP using doll and other materials. Investigator was telling a story about a child who had undergone chemotherapy, and children repeated the story by themselves	Exploratory descriptive study Observation and interview	After TP, children showed more positive behaviors, cooperating with the procedures, and working with professionals (93.3%), with relaxed posture (93.3%), establishing a bond of trust with the professional (76.6%), and smiling while playing (70%)
Arvidsson, 2016 [43] Baggott, 2006 [58] Ruland, 2007 [52] Tsimicalis, 2017 [54] Tsimicalis, 2018 [55]	Together with a self-selected avatar, the child sets out on a virtual journey from island to island (5 islands in total: “At the hospital,” “About managing things,” “My body,” “Thoughts and feelings,” and “Things one can be afraid of”)	Interactive computer-based communication tool with spoken texts, sounds, animations, and intuitively meaningful metaphors and pictures to represent symptoms and problems	Children with cancer (6–12 years old)	Not specified	Not reported	Not reported	Stage 1: translated original version of Sisom (Norwegian) into Swedish Stage 2: understanding evaluation by healthy children and pediatric nurses Stage 3: interactive low- and high-fidelity evaluations	This study was a feasibility study and did not assess the health outcomes
Beltran, 2013 [57]	Knowledge-based minigames that enabled children to learn what constitutes desired behavior Goal-setting activities Problem-solving routines to enable children to determine Motivational statements tailored to a child’s values to enhance the child’s desire to make the goal-related lifestyle changes Games to enable children to select appropriate portions and aerobic-strength-enhancing physical activities	Video games used state-of-the-art software and three-dimensional computer graphics	Preadolescents with cancer and survivors (9–12 years old)	Not specified	Not reported	Computer games are played on computers loaned to the participants at their home. However, there was no detailed description on how to use the tool	Use-experience qualitative study	This study was a feasibility study and did not assess the health outcomes
Bisignano, 2006 [33]	Compact disc read-only memory (CD-ROM) was designed to help children learn about the medical procedure, includes four components: education/information, preprocedural preparation (video modeling), breathing exercises, and distracting imagery	CD-ROM, “Spotlight on IVs”	Children with hematological or oncological diagnosis (7–18 years old)	Not specified	Not reported	Participants had approximately 20 min to instruct on how to use the computer and CD-ROM.	Randomized controlled trial (RCT)	CD-ROM reduced threat appraisals as measured by Threat Appraisal Questionnaire (TAQ) (intervention: mean ± standard deviation (SD) 83.50 ± 26.53 than at baseline mean ± SD 88.93 ± 22.92. While there was no significant difference in the control group (control: mean ± SD 90.81 ± 28.22 baseline, mean ± SD 93.38 ± 23.09. CD-ROM improved cognitive restructuring (KIDCOPE) t(21.973) = 2.38, *p* < 0.05 There was no clear difference in fear (Children’s Fear Self-Report), behavioral distress (Procedural Behavioral Rating Scale (PBRS)), and pain (Children’s Pain Self-Report).
Fazalniya, 2017 [35]	Hero and difficult struggle, championship is not related to hair, everything is calm, tales of lethargy and fatigue, tales of nausea and loss of appetite, inside of the body which contained healthy and unhealthy cells, and side effects of chemotherapy	Educational entertainment computer game, “The City of Dreams”	Not specified must be children with cancer	Not specified	Not reported	A training session was held for the children and parents regarding the content of the computer game, how to load it, and the entire process of installing and using the software. Then, to ensure that the children and parents had learned the mentioned steps, they were asked to perform the steps for the researcher	RCT	Cancer-related computer game increased quality of life (QOL), the Pediatric Quality of Life Inventory (PedsQL) 3.0 Cancer Module children self-report, compared with standard care after the intervention (intervention group: mean ± SD, 51.10 ± 18.80, control group: 43.10 ± 14.70), *p* = 0.020), and 4 weeks after the intervention (intervention group: mean ± SD, 64.70 ± 13.90, control group: 45.20 ± 13.80, *p* < 0.001)
Frygner-Holm, 2020 [44]	First story stem was based on imagination, the second was based on affect, and the third was medical play made up from variety of situations commonly experienced by children undergoing treatment for cancer	Pretend play using a variety of medical play toys and nonmedical play toys	Children with cancer (4–10 years old)	A project of international collaboration	Not reported This intervention needs the play facilitator	The play facilitator and child were alone in the room and they instructed children Pretend play consisted of six to eight 25–35 min sessions.	Mixed method	Pretend play increased self-efficacy (developed scale) for all participants after the play intervention Three patients did not change the Health-Related QOL score (Generic Health-Related Quality of Life (HRQOL)) and one increased There was no adverse event or increased worrying
Fuemmeler, 2020 [45]	Application (app) includes (1) an app and backend administrative dashboard; (2) brief phone meetings with a health coach; and (3) educational print materials for each child and parent	Smartphone applications, “The Mila Blooms”	Childhood survivors, aged 12–17 years old	Not specified	Not reported	There was a description about usage for the study participants. However, there was no description for general	Quasi-experimental single-group pretest/posttest	App decreased moderate to vigorous physical activity (triaxial accelerometers (ActiGraph GT3X+ activity monitor)) from pre to post App increased sedentary activity and fruits and vegetables self-efficacy (The PACE Adolescent Psychosocial Measures) from pre to post
Jones, 2010 [36]	Although there was a description about recommendation from adolescents, parents, and healthcare professionals, there was no detailed description	CD-ROM	Adolescents with solid tumors (12–18 years old)	Consulting company and healthcare professionals	Not reported	The user can navigate easily from one area to another throughout the CD-ROM, using TV screens or menus. A glossary is included to explain specific terms (highlighted in the text), and games are included throughout the CD-ROM	RCT	CD-ROM improved Health Locus of Control (Wallston Multidimensional Health Locus of Control Scale B (MHLC-B)) compared with Handbook group (*t*-value, 2.479, df = 63, *p* = 0.016). There was no significant group difference on QOL (Pediatric Oncology Quality of Life Scale: POQOLS), Self-efficacy (degree of confidence to perform), coping (KIDCOPE (Older Version)), or Cancer Knowledge measures (developed questionnaire) between pre-post scores.
Kato, 2008 [37] Beale, 2006 [60] Beale, 2007 [59] Kato, 2006 [61] Kurt, 2013 [38]	Destroying cancer cells and managing common treatment-related adverse effects such as bacterial infections, nausea, and constipation by using chemotherapy, antibiotics, antiemetics, and a stool softener as ammunition	Personal computer game, “Re-Mission”	Adolescents and young adults, aged 13–29 years old	HOPELAB: a team of behavioral scientists, designers, impact investors, and digital tech experts	www.re-mission.net (accessed on 20 September 2022)	The players control a nanobot, “Roxxi,” in three-dimensional environments within the bodies of young patients with cancer. However, there was no detail description on how to use the tool	RCT	Cancer-related computer game increased in antibiotic adherence (oral TMP/SMX, MEMS-cap monitoring) (intervention group: mean ± SD, 34.4 ± 2.5 doses, control group: 29.5 ± 2.6 doses, *p* = 0.012), cancer-related knowledge (Cancer Knowledge Scale) (*p* = 0.035), and cancer-specific self-efficacy (developed Self-efficacy Scale) (*p* = 0.011) There was no difference on general treatment adherence (Chronic Disease Compliance Instrument (CDCI)), oral chemotherapy adherence (6MMP concentrations), QOL (Pediatric Quality of Life self-report instrument (PQL)), perceived stress scale, and health locus of control (Multidimensional Health Locus of Control Scale Form C) between cancer-related computer game group and no cancer-related game group
Li, 2011 [39]	A variety of group playing activities, in particular, involves using virtual reality through interactive simulations created by computer hardware and software to present children to engage in environments that appear and feel similar to re-al-work objects and events	Therapeutic play (TP) using virtual reality computer games by research nurses in the playroom	Children with cancer (8–16 years old), were undergoing active treatment	Not specified	Not reported	Not reported	Pre- and post- test with control group	TP reduced depression (Center for Epidemiologic Studies Depression Scale for Children (CES-DC)) on day 7 compared with usual care (therapeutic group: 20.60, control group: 25.97) There was no clear difference in anxiety (short form of the Chinese Version of the State Anxiety Scale for Children (CSAS-C)) between therapeutic play and usual care (therapeutic group: 19.48, control group: 21.06)
Linder, 2021 [48]	The app supports the report of the prevalence, severity, and associated bother of eight general symptoms: pain, nausea, vomiting, fatigue, difficulty sleeping, appetite changes, coughing, and dizziness.	Game-based symptom-reporting app, “Color Me Healthy”	Children with cancer (6–12 years old), were undergoing active treatment	Not specified; however, children and clinicians provided input regarding symptoms	Not reported	Children receive up to two daily rewards: one for logging into the app and a second for completing key daily tasks within the app. However, there was no detailed description of how to use the tool	Verification of children’s app usage and interview survey	This study was a feasibility study. Thus, there was no assessment of health outcomes
O’Conner-Von, 2009 [50]	Core components of the program include information about (a) cancer, (b) cancer treatment, (c) feelings about having cancer, (d) dealing with friends and school, (e) healthy coping strategies, and (f) advice from the adolescent cancer experts	Web-based educational program “Coping with Cancer”	Children with cancer (10–16 years old)	Not specified	Not reported	Not reported	Not assessed, but they planned a field test of the program next	This study was a feasibility study. Thus, there was no assessment of health outcomes
Pitillas, 2018 [51]	There are components related to these aims. (1) Reality testing and ego strengthening, and (2) unveiling and working through unconscious conflicts related to disease, and (3) defense maturation and problem solving	Psychoanalytic PT	Children with cancer (18 months-14 years old)	Not specified	Not reported	Not reported	Not reported	This study just described play therapy. Thus, there was no report on the impact of communication tool on health outcomes
Sajjad, 2014 [40]	The main theme is that the patient hits the enemy character through the powerful use of weapons (white blood cells). The enemy character (a brain tumor) is targeted and destroyed, increasing the patient’s health bar.	3D Graphical Imagery Therapy game	Children with a brain tumor, (10–14 years old)	Not specified	Not reported	The clinical psychologist instructed the game to the patients. However, there was no detailed description of how to use the tool	Quasi-experimental controlled pretest/posttest	The 3D game improved self-conceptualization (Beck Self Concept Inventory For Youth) (Intervention group: 76.4%, control group: 53.2%) The 3D game reduced anxiety (Beck Anxiety Inventory For Youth) (Intervention group: 35.5%, control group: 48.6%), depression (Beck Depression Inventory For Youth) (Intervention group: 39.0%, control group: 49.4%), anger (Beck Anger Inventory For Youth) (Intervention group: 40.9%, control group: 43.3%), and disruptive behavior (Beck Disruptive Behavior inventory For Youth) (Intervention group: 48.1%, control group: 51.6%)
Sposito, 2016 [53]	1. The making of the puppet by the child, followed by the child interview using the puppets. 2. The use of puppets as a playful strategy during the interviews with hospitalized children with cancer was structured	Interviews (54–71 min) using puppets	Children with cancer (7–12 years old)	Researcher The first author, an occupational therapist, conducted the interview.	Not reported	Making of the puppet and following the child’s interview using the puppets	Use-experience qualitative study	The use of puppets facilitated the children’s expression of feelings and their verbal communication. Children could express their experiences in the hospital to the researchers.
Tyc, 2003 [41]	Educational video that discussed the short- and long-term physical and social consequences of tobacco use; late effects risk counseling focused on potential chemotherapy and radiation treatment-related toxicities that can be exacerbated by tobacco use and the survivors’ increased vulnerability to tobacco-related health risks relative to their healthy peers	Educational video to reduce intentions to use tobacco among pediatric cancer survivors, Qualitative study	Preadolescents and adolescents with cancer (Not specified the ages)	Not specified	Not reported	A master’s level psychologist provided the intervention over 50–60 min, and a trained research nurse conducted the follow-up telephone counseling. However, there was no detailed description of how to use the tool	RCT	Risk counseling intervention improved knowledge related to the adverse consequences associated with tobacco use (intervention group: mean ± SD, 24 ± 1.4, standard care group: 22.7 ± 2.4), perceived vulnerability to tobacco-related health risks (Intervention group: mean ± SD, 35.9 ± 4.6, standard care group: 32.5 ± 5.7), and decreased intention to use tobacco (intervention group: mean ± SD, 7.8 ± 4.0, standard care group: 10.0 ± 3.9) at 12 months (*p* = 0.002). There was no clear difference in knowledge, perceived vulnerability, and intention at 6 months.
Wiener, 2011 [56]	ShopTalk consists of a colorful board with ten stores, each with a set of 15 question cards related to the theme of the individual store (150 questions total)	Therapeutic game, “Shop Talk”	Children with cancer (7–16 years old)	Researcher	It was distributed for the pilot study. However, there was no description of the access to general	Players roll the dice to move their “shopping bag” piece around the board, attempting to enter each store, at which point they become a “customer” and are asked a question by another player. However, there was no detailed description of how to use the tool	They planned a randomized controlled trial as a next step	This study was a feasibility study. Thus, there was no assessment of health outcomes
**For children and their families**								
Dragone, 2002 [34]	The Get Better Place (research studies, medicines, treatment, health care team), Help Yourself (areas in which children can exert some control, including nutrition, preventing infections, pain control, creative arts, and relaxation techniques), The Testing Center (bone marrow tests and spinal taps, blood tests, radiology tests, heart testing, and vital signs), The Filland Fly (red blood cells, white blood cells, and platelets), The Space Mall (changes in appearance, central venous catheters, anatomy and physiology, and resource/reference section), and The Movies (video hospital tour, living with leukemia, expert explanation of leukemia, and siblings’ views of leukemia)	CD-ROM, “Kidz with Leukemia: A Space Adventure”	Children with leukemia, (4–11 years old) and their families	Healthcare professionals	Not reported	The intervention group received the CD-ROM, Kidz with Leukemia: A Space Adventure, to use for approximately 3 months. However, there was no detailed description on how to use the tool	RCT	Children in the CD-ROM group, compared with those in the book group, showed increased feelings of control over their health (leukemia children’s health locus of control (LCHLC)).
Greenspoon, 2019 [46]	Relevant anatomy, physiology of ovulation, egg retrieval, and process of cryopreservation	7-minute whiteboard video with hand-drawn sketches in full color	Patients with cancer, (13–39 years old) and parents	Not specified	Not reported	Not reported	Questionnaires survey	There was an association between younger age and greater improvement in general knowledge scores on fertility preservation (*p* = 0.007; r = −0.26)
Kock, 2015 [47]	Disease, a reminder service for follow-up examinations, and a calendar function to coordinate these examinations	Android mobile application	Children with childhood cancer (>15 years old) and their relatives	Not specified	Not reported	Not reported	Questionnaire survey: usability questionnaires following the ISO 9241/110 norm	This study was a feasibility study. Thus, there was no assessment of health outcomes
Murphy, 2012 [49]	Cancer-related infertility and the options available for pediatrics based on available literature and existing brochures from Moffitt Cancer Center, Fertile Hope, and the Onco-fertility Consortium	Gender concordant brochures	Pediatric oncology patients and parents (Ages not specified)	Not specified	Not reported	Not reported	Interview survey	This study was a feasibility study. Thus, there was no assessment of health outcomes
**For healthcare professionals**								
No communication tool was identified.								

Footnote: the background is used for visibility. CDCI: Chronic Disease Compliance Instrument, CD-ROM: compact disc read-only memory, CES-DC: Center for Epidemiologic Studies Depression Scale for Children, CSAS-C: Chinese Version of the State Anxiety Scale for Children, HRQL: Health-Related Quality of Life, LCHLC: leukemia children’s health locus of control, MHLC-B: Multidimensional Health Locus of Control Scale B, TAQ: Threat Appraisal Questionnaire TP: therapeutic play, PedsQL: the Pediatric Quality of Life Inventory, PBRS: Procedural Behavioral Rating Scale, POQOLS: Pediatric Oncology Quality of Life Scale, PQL: Pediatric Quality of Life self-report instrument, RCT: randomized controlled trials, SD: standard deviation.

#### 3.3.1. Communication Tools with Children with Cancer

We found 21 communication tools that provide cancer-related information to children with cancer. Of these, 17 tools targeted children with cancer, and four targeted children and their families. There was no communication tool targeting the HCPs or all of these populations. Although most of the communication tools focused on coping with cancer and included provision of information and education, their contents varied. The main contents of communication tools were related to procedures and treatments [33,37,39,40,42,44], problem solving [51,57], fertility [46,49], disease [56], symptom management [48], tobacco use [41], reminders for examinations and a calendar [47], and multiple kinds of content [34,35,43,45,50]. One study was interviewed based on patients’ experiences [53] and one did not describe the contents of the communication tools [36].

Most of the tools incorporated elements of play. Fifteen tools were computer based [33,34,35,36,37,39,40,41,43,45,46,47,48,50,57], four involved play therapy [42,44,51,56], one was a brochure [49], and one was an interview [53].

Nine studies specified the target population as children and adolescents with cancer undergoing treatments [33,39,40,43,44,49,53,56,57], and four studies included survivors [45,47,49,57]. Three studies included preschool children [34,44,51], 11 studies included school children [34,39,40,41,43,44,48,51,53,56,57], and 11 studies included adolescents [33,36,37,39,40,41,45,46,50,51,56]. Although most studies did not describe developers, three were developed by HCPs [34,36,44], two by researchers [53,56], one by a team of professionals [37], and one was provided input regarding symptoms by children and clinicians [48].

#### 3.3.2. How to Use Communication Tools

Only one study specified access to the computer game (www.re-mission.net) (accessed on 20 September 2022) [37]. It is accessible to everyone free of charge. Five studies provided instructions regarding how to use the communication tools by facilitators, including psychologists [33,35,36,40,44]. Therapeutic play using a doll was conducted by nurses [42] and therapists [53]. Other studies did not mention the usage instructions. There was no tool that had accompanying instructions for families and HCPs.

#### 3.3.3. How to Validate and Evaluate Communication Tools

Nine studies assessed the effects of communication tools using experimental study design and six of them were randomized controlled studies [33,34,35,36,37,41]. The feasibility of communication tools was evaluated qualitatively by eight studies [42,43,48,49,50,51,53,57], quantitatively by four studies [45,46,47,56], and mixed by one study [44].

#### 3.3.4. The Impacts of Communication Tools on Health Outcomes

Experimental studies showed that communication tools improved health outcomes for children with cancer. CD-ROM reduced threat appraisals, improved cognitive restructuring coping [33], and increased the health locus of control [34,36]. Computer games improved adherence, self-efficacy, knowledge [37], quality of life [35,38], and self-conceptualization [40]. Therapeutic play using virtual reality reduced children’s depression [39]. Video and risk counseling intervention improved knowledge, perceived vulnerability, and decreased intention [41]. Although there was little evidence for behavior change attributable to the communication tools, feasibility studies showed that the communication tools might be a feasible way to communicate with children about cancer-related information [42,43,44,45,46,47,48,49,50,51,52,53,54,55,56,57].

## 4. Discussion

### 4.1. Summary of Evidence

This scoping review identified 25 primary studies that evaluated the feasibility and effectiveness of communication tools. The studies in question were published between 2002 and 2021. Our review mapped existing communication tools and found 21 tools that provide cancer-related information to children with cancer. Experimental studies included in this review showed that communication tools might improve children’s knowledge and may have positive psychological effects as a result of sharing cancer-related information interactively [33,34,35,36,37,39,40,41]. Due to limited guides [9,11] and well-designed training focused on healthcare communication for children [62], communication tools might help families and HCPs communicate cancer-related information to children. We have identified the types of communication tools that are lacking, and four areas that should be enhanced in future practice and research. There is a lack of communication tools that are (1) accessible and validated, (2) designed for HCPs, (3) target children, families, and HCPs, and (4) are designed to meet the needs of children and families.

First, accessible and validated communication tools are needed. Ranmal et al. 2008 suggested that interventions to enhance communication with children with cancer have not been widely and rigorously evaluated [25]. Even though more than ten years have passed since that systematic review was conducted, the results of this study also showed a lack of research that evaluated the effectiveness of communication tools. Additionally, the tools available were limited, and most studies did not describe how to use them. This gap might be due to the difficulty of including children in the study. When involving children in research, various factors can cause harm, such as stress due to participation in the study, revealing hidden or suppressed feelings and memories, expressing concerns, and worries about sharing [63]. Of course, research should be considered with regard to the ethical principles and issues of involving children [64]. At the same time, research involving children and families is needed to evaluate how communication tools are available and how they support children.

Second, communication tools to guide HCPs are needed. HCPs must show empathy for their patients and families [65]. However, telling a child they have a life-threatening illness can be burdensome for HCPs [66]. Recently, the complexity of communication with life-threatened children has been pointed out, and the need for research and guides on communication has been appealed [9]. To build child- and family-centered communication, it is suggested to follow a guide to communication strategies based on rigorous communication science [67]. Further research is needed to develop communication tools to guide HCPs and report the detail of how to use them to communicate with children with cancer.

Third, communication tools that target children, families, and HCPs are needed. Children with cancer could regain safety and control based on their knowledge about their bodies, cancer, and treatments [68]. Therefore, HCPs should communicate cancer-related information with children understandably, considering children’s developmental stages. Nijhof et al., also argued that stimulating play behavior leads children with chronic illnesses to adapt to stressful conditions and promotes the development of emotional, cognitive, and social [69]. Communication aims to deliver information as well as to support children and families’ coping and well-being. We found communication tools that introduced play elements, such as play therapy and computer games. These tools might not only help children understand their condition and cope with cancer, but may also promote their development. However, we did not find any tools that can be used commonly by all three populations: children with cancer, their families, and HCPs. Communication is a basic component needed in order to build a positive relationship among patients, families, and HCPs, resulting in the delivery of quality care [70]. Thus, we should consider the interaction of all these population, not just children, to communicate with children effectively. Further research is needed to develop and evaluate communication tools that target children, families, and HCPs.

Fourth, communication tools that are developed and evaluated to meet the needs of children and their families are needed. Previous research has indicated children have specific needs for sharing information, and different views are held among children, their parents, and HCPs [9,15]. Although children might need to know cancer-related information immediately at diagnosis, parents might control the flow information to their children due to their own emotional distress and belief [9,15]. If children perceive communication as parent centered, they might be disempowered. In contrast, children can be empowered to cope with cancer when they feel that HCPs address their information and developmental needs [68]. HCPs should understand families’ struggles and collaborate with them to respect children’s opinions. In this review, we found only a few tools developed involving children’s opinions. People-centered health services are fundamental in healthcare [71]. Future research is needed to develop and evaluate communication tools that meet the needs of children and their families to enhance child-centered communication.

### 4.2. Strengths and Limitations

This review was conducted following the protocol to avoid the potential risk of bias. Moreover, we reported this review following PRISMA-ScR to improve its completeness and transparency. However, our scoping review has some limitations. First, this review searched in title only to identify the studies which met our inclusion criteria. We believe that these studies were sufficient to map the current situation. However, it is undeniable that some potential studies may have been overlooked. Second, due to the limited number of included studies, we could not classify the communication tools by age. Third, we did not assess the risk of bias or critical appraisal. Therefore, our research results might contain potential bias related to the included studies.

### 4.3. Implications for Practices and Future Research

Although the evidence is limited and communication tools might not apply to every situation, they might be useful and helpful in communicating cancer-related information with children. Still there are a variety of contents and types of communication tools, and HCPs should use them with consideration of whether they are appropriate for each child and family.

Further research is needed to develop and evaluate communication tools, which are (1) accessible and validated, (2) designed for HCPs, (3) target children, families, and HCPs, and (4) are designed to meet the needs of children and families. We also recommend simultaneously investigating the children’s and families’ experiences of using communication tools to understand how they support children. This would lead to deeper insights. Moreover, Future research is needed to focus on how HCPs communicate cancer-related information with children and report the detail of communication tools to be utilized in practice.

## 5. Conclusions

This scoping review aimed to map the existing communication tools that provide cancer-related information to children with cancer. Communication tools might support HCPs in providing effective communication and may positively impact how children and families cope with cancer. However, there is a lack of communication tools that are (1) accessible and validated, (2) for HCPs, (3) target children, families, and HCPs, and (4) are designed to meet the needs of children and families. Further research is needed to develop and evaluate these communication tools. Moreover, it is necessary to investigate how communication tools support children, their families, and HCPs.

## Supplementary Materials

The following supporting information can be downloaded at: https://www.mdpi.com/article/10.3390/cancers14194624/s1.
