# Supplementary material for: Communication Tools Used in Cancer Communication with Children: A Scoping Review
Source: Cancers (Basel). 2022 Sep 23;14(19):4624. doi: 10.3390/cancers14194624 (PMC9563078; doi:10.3390/cancers14194624)
Supplement: Supplementary file 1 [file cancers-14-04624-s001.zip › cancers-1918563-supplementary.pdf]

Supplementary file: Search Strategies

A. CENTRAL

Advanced Search/Search Manager

#1 ((Child* or Preschool* or Adolescen* or Teen* or Young or Youth* or Schoolchild* or Pediatric* or Paediatric* or Boys or Girls) and (Cancer* or Carcinoma* or Tumour* or Tumor* or Malignan* or Metasta* or Neoplas* or Leukaemi* or Leukemi* or Lymphoma* or Sarcoma* or Carcinoma* or Oncolog* or Radiotherap* or "Radio Therap*" or Chemotherap* or Lifethreatening or "Life Threatening" or Terminally Ill or Terminal Illness* or Terminal Condition* or "Terminal Care" or Palliative or Hospice* or "End of Life") and (Communicat* or Video* or Audio* or Multimedi* or Multi-Media or Hypermedia or Book or Booklet* or Comic* or Books or Fiction* or Manga* or Pamphlet* or Brochure* or Movie* or Film* or Motion Picture* or Cinema* or CD-ROM or DVD or Compact Disk* or Compact Disc* or Game or Games or Gaming or Internet or Web or Website* or Computer or Play or Plays or Roleplay* or Story* or Stories or Song or Songs or Sing or Singing or Tale or Tales or Toy or Toys or App or Apps or Mobile Application* or Phone Application* or Smartphone Application* or iPhone Application* or Disclos* or Decision Making or Bad News or Doll or Dolls or Art or Arts or Music* or Painting* or Drawing*)):ti in Trials 178

B. CINAHL

Advanced Search

S1 TI (Child* OR Preschool* OR Adolescen* OR Teen* OR Young OR Youth* OR Schoolchild* OR Pediatric* OR Paediatric* OR Boys OR Girls) AND (Cancer* OR Carcinoma* OR Tumour* OR Tumor* OR Malignan* OR Metasta* OR Neoplas* OR Leukaemi* OR Leukemi* OR Lymphoma* OR Sarcoma* OR Carcinoma* OR Oncolog* OR Radiotherap* OR "Radio Therap*" OR Chemotherap* OR Lifethreatening OR "Life Threatening" OR Terminally Ill OR Terminal Illness* OR Terminal Condition* OR "Terminal Care" OR Palliative OR Hospice* OR "End of Life") AND (Communicat* OR Video* OR Audio* OR Multimedi* OR Multi-Media OR Hypermedia OR Book OR Booklet* OR Comic* OR Books OR Fiction* OR Manga* OR Pamphlet* OR Brochure* OR

Movie* OR Film* OR Motion Picture* OR Cinema* OR CD-ROM OR DVD OR Compact Disk* OR Compact Disc* OR Game OR Games OR Gaming OR Internet OR Web OR Website* OR Computer OR Play OR Plays OR Roleplay* OR Story* OR Stories OR Song OR Songs OR Sing OR Singing OR Tale OR Tales OR Toy OR Toys OR App OR Apps OR Mobile Application* OR Phone Application* OR Smartphone Application* OR iPhone Application* OR Disclos* OR Decision Making OR Bad News OR Doll OR Dolls OR Art OR Arts OR Music* OR Painting* OR Drawing*)
Limiters - Exclude MEDLINE records 512

C. Embase

1 ((Child* or Preschool* or Adolescen* or Teen* or Young or Youth* or Schoolchild* or Pediatric* or Paediatric* or Boys or Girls) and (Cancer* or Carcinoma* or Tumour* or Tumor* or Malignan* or Metasta* or Neoplas* or Leukaemi* or Leukemi* or Lymphoma* or Sarcoma* or Carcinoma* or Oncolog* or Radiotherap* or "Radio Therap*" or Chemotherap* or Lifethreatening or "Life Threatening" or Terminally Ill or Terminal Illness* or Terminal Condition* or "Terminal Care" or Palliative or Hospice* or "End of Life") and (Communicat* or Video* or Audio* or Multimedi* or Multi-Media or Hypermedia or Book or Booklet* or Comic* or Books or Fiction* or Manga* or Pamphlet* or Brochure* or Movie* or Film* or Motion Picture* or Cinema* or CD-ROM or DVD or Compact Disk* or Compact Disc* or Game or Games or Gaming or Internet or Web or Website* or Computer or Play or Plays or Roleplay* or Story* or Stories or Song or Songs or Sing or Singing or Tale or Tales or Toy or Toys or App or Apps or Mobile Application* or Phone Application* or Smartphone Application* or iPhone Application* or Disclos* or Decision Making or Bad News or Doll or Dolls or Art or Arts or Music* or Painting* or Drawing*)).ti. (1570)

2 limit 1 to embase (754)

D. PsycINFO

1 ((Child* or Preschool* or Adolescen* or Teen* or Young or Youth* or Schoolchild* or Pediatric* or Paediatric* or Boys or Girls) and (Cancer* or Carcinoma* or Tumour* or Tumor* or Malignan* or Metasta* or Neoplas* or Leukaemi* or Leukemi* or Lymphoma* or Sarcoma* or

Carcinoma* or Oncolog* or Radiotherap* or "Radio Therap*" or Chemotherap* or Lifethreatening or "Life Threatening" or Terminally Ill or Terminal Illness* or Terminal Condition* or "Terminal Care" or Palliative or Hospice* or "End of Life") and (Communicat* or Video* or Audio* or Multimedi* or Multi-Media or Hypermedia or Book or Booklet* or Comic* or Books or Fiction* or Manga* or Pamphlet* or Brochure* or Movie* or Film* or Motion Picture* or Cinema* or CD-ROM or DVD or Compact Disk* or Compact Disc* or Game or Games or Gaming or Internet or Web or Website* or Computer or Play or Plays or Roleplay* or Story* or Stories or Song or Songs or Sing or Singing or Tale or Tales or Toy or Toys or App or Apps or Mobile Application* or Phone Application* or Smartphone Application* or iPhone Application* or Disclos* or Decision Making or Bad News or Doll or Dolls or Art or Arts or Music* or Painting* or Drawing*)).ti. (447)

E. PubMed

(Child*[TI] OR Preschool*[TI] OR Adolescen*[TI] OR Teen*[TI] OR Young[TI] OR Youth*[TI] OR Schoolchild*[TI] OR Pediatric*[TI] OR Paediatric*[TI] OR Boys[TI] OR Girls[TI]) AND (Cancer*[TI] OR Carcinoma*[TI] OR Tumour*[TI] OR Tumor*[TI] OR Malignan*[TI] OR Metasta*[TI] OR Neoplas*[TI] OR Leukaemi*[TI] OR Leukemi*[TI] OR Lymphoma*[TI] OR Sarcoma*[TI] OR Carcinoma*[TI] OR Oncolog*[TI] OR Radiotherap*[TI] OR "Radio Therap*"[TI] OR Chemotherap*[TI] OR Lifethreatening[TI] OR "Life Threatening"[TI] OR Terminally Ill[TI] OR Terminal Illness*[TI] OR Terminal Condition*[TI] OR "Terminal Care"[TI] OR Palliative[TI] OR Hospice*[TI] OR "End of Life"[TI]) AND (Communicat*[TI] OR Video*[TI] OR Audio*[TI] OR Multimedi*[TI] OR Multi-Media[TI] OR Hypermedia[TI] OR Book[TI] OR Booklet*[TI] OR Comic*[TI] OR Books[TI] OR Fiction*[TI] OR Manga*[TI] OR Pamphlet*[TI] OR Brochure*[TI] OR Movie*[TI] OR Film*[TI] OR Motion Picture*[TI] OR Cinema*[TI] OR CD-ROM[TI] OR DVD[TI] OR Compact Disk*[TI] OR Compact Disc*[TI] OR Game[TI] OR Games[TI] OR Gaming[TI] OR Internet[TI] OR Web[TI] OR Website*[TI] OR Computer[TI] OR Play[TI] OR Plays[TI] OR Roleplay*[TI] OR Story*[TI] OR Stories[TI] OR Song[TI] OR Songs[TI] OR Sing[TI] OR Singing[TI] OR Tale[TI] OR Tales[TI] OR Toy[TI] OR Toys[TI] OR App[TI] OR Apps[TI] OR Mobile Application*[TI] OR Phone Application*[TI] OR Smartphone Application*[TI] OR iPhone Application*[TI] OR

Disclos*[TI] OR Decision Making[TI] OR Bad News[TI] OR Doll[TI] OR Dolls[TI] OR Art[TI] OR Arts[TI] OR Music*[TI] OR Painting*[TI] OR Drawing*[TI]) 1073

a
